# Reliability, Validity and Usefulness of 30–15 Intermittent Fitness Test in Female Soccer Players

**DOI:** 10.3389/fphys.2016.00510

**Published:** 2016-11-17

**Authors:** Nedim Čović, Eldin Jelešković, Haris Alić, Izet Rađo, Erduan Kafedžić, Goran Sporiš, Daniel T. McMaster, Zoran Milanović

**Affiliations:** ^1^Faculty of Sport and Physical Education, University of SarajevoSarajevo, Bosnia and Herzegovina; ^2^Institute of KinesiologySarajevo, Bosnia and Herzegovina; ^3^Faculty of Kinesiology, University of ZagrebZagreb, Croatia; ^4^Sport Performance Research Institute New Zealand, AUT UniversityAuckland, New Zealand; ^5^Health, Sport and Human Performance, University of WaikatoHamilton, New Zealand; ^6^Faculty of Sport and Physical Education, University of NišNiš, Serbia

**Keywords:** 30–15 intermittent fitness test, aerobic, cardiorespiratory fitness, intermittent activity, soccer, high intensity interval training

## Abstract

**PURPOSE:** The aim of this study was to examine the reliability, validity and usefulness of the 30–15_IFT_ in competitive female soccer players.

**METHODS:** Seventeen elite female soccer players participated in the study. A within subject test-retest study design was utilized to assess the reliability of the 30–15 intermittent fitness test (IFT). Seven days prior to 30–15_IFT_, subjects performed a continuous aerobic running test (CT) under laboratory conditions to assess the criterion validity of the 30–15_IFT_. End running velocity (V_CT_ and V_IFT_), peak heart rate (HRpeak) and maximal oxygen consumption (VO_2max_) were collected and/or estimated for both tests.

**RESULTS:** V_IFT_ (ICC = 0.91; CV = 1.8%), HRpeak (ICC = 0.94; CV = 1.2%), and VO_2max_ (ICC = 0.94; CV = 1.6%) obtained from the 30–15_IFT_ were all deemed highly reliable (*p* > 0.05). Pearson product moment correlations between the CT and 30–15_IFT_ for VO_2max_, HRpeak and end running velocity were large (*r* = 0.67, *p* = 0.013), very large (*r* = 0.77, *p* = 0.02) and large (*r* = 0.57, *p* = 0.042), respectively.

**CONCLUSION:** Current findings suggest that the 30–15_IFT_ is a valid and reliable intermittent aerobic fitness test of elite female soccer players. The findings have also provided practitioners with evidence to support the accurate detection of meaningful individual changes in V_IFT_ of 0.5 km/h (1 stage) and HRpeak of 2 bpm. This information may assist coaches in monitoring “real” aerobic fitness changes to better inform training of female intermittent team sport athletes. Lastly, coaches could use the 30–15_IFT_ as a practical alternative to laboratory based assessments to assess and monitor intermittent aerobic fitness changes in their athletes.

## Introduction

Female soccer has increased in popularity and participation over the past 20 years; as a result the skill level and physical demands of completion have also increased. The main characteristics of female and male soccer are similar in that match activity, aerobic power, sprinting capacity and exercise performance vary between playing positions (Rhodes and Mosher, 1992; Krustrup et al., 2005; Nikolaidis, 2014b). In addition, the physical profiles of the female soccer players defers between levels of competition; where elite players are faster, more powerful and have a greater aerobic capacity in comparison to non-elite players (Nikolaidis, 2010; MK Tood and Chisnal, 2013). Krustrup et al. (2005) has shown that average heart rate (HR) during matches was 87% of HRmax, with HRpeak values reaching 97% HRmax during high intensity running (HIR) efforts. Of interest, the duration of and ability to repeat HIR was highly correlated with aerobic capacity (VO_2max_), specifically in last 15 min of each half (Krustrup et al., 2005). However, HRpeak was poorly correlated with HIR; these findings support the notion that training prescription in female soccer should be based on individual high intensity intermittent aerobic fitness and not HRmax.

An apparent misinterpreted physiological response to intermittent high intensity interval training (HIIT) has emerged, as a result of negligence and a lack of understanding the information obtained from valid intermittent aerobic fitness tests (Buchheit, 2010). There are a number of field based fitness tests that attempt to predict aerobic capacity with varying levels of accuracy, including: the Montreal Track Test (Uger and Boucher, 1980); Yo-Yo Intermittent Recovery Test Level 1 (IR1) (Castagna et al., 2006; Dupont et al., 2010); and the multi-stage fitness test (Léger et al., 1988). A limitation with most of these aerobic fitness test is that athletes with lower maximal running speeds are required to perform supramaximal (>120% of aerobic capacity) high intensity efforts with directional changes at the same pace as faster athletes; and in turn are utilizing a higher proportion of their anaerobic speed reserve (Thomas et al., 2015).

For the purpose of resolving training intensity prescription issues in intermittent team sports, the 30–15 Intermittent Fitness Test (30–15_IFT_) was developed (Buchheit, 2008; Haydar et al., 2011). The 30–15_IFT_ estimates aerobic capacity (VO_2max_), determines maximal heart rate (HRmax) and anaerobic and intermittent HIR capacity (Buchheit and Rabbani, 2014; Thomas et al., 2015). The primary outcome measure of the 30–15_IFT_ is running velocity (V_IFT_)for the last completed stage (Buchheit, 2010), a suitable alternative to vVO_2max_ and HRpeak (Rabbania and Buchheita, 2015). As demonstrated, running speed at maximal oxygen uptake (vVO_2max_) in continuous straight-line cardiorespiratory fitness tests is much lower than V_IFT_, implying that anaerobic metabolism engagement is much higher in the 30–15_IFT_ (Buchheit, 2010). Lactic acid was up to 40% greater following the 30–15_IFT_ in comparison to theLéger-Boucher track test (Buchheit et al., 2009a; Buchheit, 2010). In addition, V_IFT_ is highly correlated (*r* = 0.80) to other intermittent fitness tests (e.g., Léger-Boucher test and Yo-Yo IR1) end speed (Buchheit, 2008). The validity of 30–15_IFT_ simultaneously reflects broad spectrum of physiological, mechanical and neuromuscular components (Buchheit, 2008).

The 30–15_IFT_ was initially validated using female handball players (Buchheit, 2008, 2010). It has since been validated for elite ice hockey (Buchheit et al., 2011), male rugby (Scott et al., 2015), male semi-professional soccer (Thomas et al., 2015), and basketball (Buchheit, 2008, 2010) players. The reliability and effectiveness of 30–15_IFT_ to monitor intermittent fitness changes was also demonstrated in the above studies. The 30–15_IFT_ is highly reliable (ICC = 0.90–0.96) across a range of sports, suggesting that a V_IFT_ change of 0.5 km/h (1 running stage) is substantial (Buchheit, 2010) for detecting “real” changes in performance. The 30–15_IFT_ is also applicable to a number of other sports including: wheelchair basketball (Weissland et al., 2015), judo, futsal, netball and field hockey (Buchheit, 2010).

To date no research has investigated the reliability and validity of the 30–15_IFT_, in comparison to a standard continuous incremental running test (CT) in elite female soccer players. Of interest is the practicality of the 30–15_IFT_ to provide coaches with a valuable aerobic fitness measure for the purpose of monitoring and determining the level of preparedness of elite female soccer players. The aim of this study was to examine the reliability, validity and usefulness of the 30–15_IFT_ in competitive female soccer players. It is expected that 30–15_IFT_ will be highly reliable and a valid indicator of aerobic fitness and HRmax; and in turn should provide meaningful intermittent fitness data (V_IFT_) for individualized high intensity interval training (HIIT) prescription.

## Methods

### Experimental approach and design

A within subject test-retest study design was utilized; where the 30–15_IFT_ was performed on two separate occasions (7 days between trials). Seven days prior to 30–15_IFT_, subjects performed a CT under laboratory conditions. The CT was used to precisely estimate VO_2max_ and HRmax. The CT was performed at the beginning of preparation period after 1 week of low intensity soccer training. The 30–15_IFT_ test-retest were performed at the same time of day (12.00–13.00). A standard indoor facility (40 × 20 m) with synthetic non-slippery surface was used for 30–15_IFT_. The subjects wore standard soccer attire including personal boots and were asked to refrain from performing any intense physical activity 48 h prior to testing.

### Subjects

Seventeen well trained (training age = 5 years) elite female soccer players (age = 22.8 ± 4.3 years; height = 164 ± 6.9 cm; body mass = 57.3 ± 9.2 kg) participated in the study. Participants were members of the state champion's soccer club; in addition eight of the subjects play for the senior national team. The subjects trained 5.4 ± 1.7 times per week (9.9 ± 2.3 h per week). All subjects were free of injury, illness and disease as determined by a medical examination prior to study participation. Seventeen players completed the initial 30–15_IFT_ and continuous running test (CT). One player was excluded from the remainder of the study due to a previous injury; and data from four of the subjects following CT were excluded due to methodological issues (one subject was removed due to the loss of transmission from the HR belt and three due to inappropriate data storage). Sixteen subjects were included for the test-retest reliability and 13 subjects for validation of the 30–15_IFT_. The study was approved by the Ethics Committee of the Faculty of Sport and Physical Education, University of Sarajevo according to the Helsinki Declaration guidelines. Participants were fully informed and signed a consent form that indicated they could withdraw from the study at any time.

### Continuous incremental running test

Each player performed the Taylor running continuous exercise protocol (Taylor et al., 1955) under laboratory conditions (~22°C room temperature). The graded CT featured running on motor driven treadmill (Cosmed, Rome, Italy) at slope angle of 1.5°. Participants performed the following lower limb dynamic stretches prior to the CT: leg swings, walking lunges, side lunges, ankle bounce, and single leg bounce. The initial stages of the CT served as the warm-up. Firstly, the subjects were monitored at speed of 3 km/h for 3 min. The velocity was than increased to 7 km/h followed by automated speed increase of 1 km/h each minute until volitional exhaustion (failure). An automated breath-by-breath respiratory system (K4b2, Cosmed, Rome, Italy) was used to analyze the gas exchange. All cardiorespiratory data (VO_2_-oxygen uptake, VCO_2_–carbon dioxide output, VT–tidal volume, VE–minute ventilation, RER–respiratory exchange ratio as well as PO_2_ and PCO_2_ tidal volume) were averaged across 5 s time intervals. Highest VO_2_ consumption obtained from four average values (20 s) was defined as the maximal oxygen uptake (VO_2max_). Heart rate was also monitored in real time at frequency of 1 Hz (Polar Electro Oy, Finland). Heart rate at VO_2_ peak represented HRpeak. Running velocity reached at VO_2peak_ presented tests end speed (V_CT_). For the purpose of ensuring maximum effort and volitional exhaustion was achieved the following criteria were implemented: HRpeak within 5% of the predicted HRmax (220-age), RER > 1.15, VE/VO_2_ < 30 and blood lactate > 8 mmol/l. Gas analyzer was calibrated according to manufacturer recommendations (Duffield et al., 2004) prior to each test.

### The 30–15 intermittent fitness test

Athletes performed a set of five dynamic stretches (leg swings, walking lunges, side lunges, ankle bounce and single leg bounce) prior to the 30–15_IFT_. The 30–15_IFT_ was performed as described previously (Buchheit, 2008). The test consists of 30 s shuttle runs interspersed with 15 s passive recovery periods. Subjects performed shuttles between two lines (40 m apart) at a given pace of pre-recorded audio beeps. The test starts at a velocity of 8 km/h and increases by 0.5 km/h for each successive 30 s stage. Players were verbally encouraged to complete as many stages as possible. The test ended, when the player (i) was totally exhausted and stopped on her own volition or (ii) if she was unable to reach the next 3-meter zone at the beep on three successive occasions. The running velocity during the last completed stage was taken as the maximum running speed (V_IFT_). Estimated VO_2maxIFT_ was calculated from V_IFT_ and the athlete's gender (G), age (A) and body mass (BM) as follows (Buchheit, 2008):

$$\begin{array}{l} {\text{VO}}_{2\text{maxIFT}}\;\left(\text{ml}/\text{min}/\text{kg}\right)=28.3-2.15\text{G}-0.741\text{A}-0.0357\text{BM}\\ +\;0.058\text{AX}{\text{V}}_{\text{IFT}}+1.03{\text{V}}_{\text{IFT}.} \end{array}$$

A video (Sony DSLR-A700) recording of the test was reviewed for cases where V_IFT_ was uncertain. Heart rate was also monitored in real time at frequency of 1 Hz (Polar Electro Oy, Finland) during each test.

### Statistical analysis

Means and standard deviations (SD) with 90% confidence interval limits (90% CI) were used to represent centrality and spread of data. Data normality was assessed using Shapiro-Wilk test he inspection of Q-Q plots and the homogeneity of the variance was verified using Levene test. Paired sample *t*-tests were used to determine if a learning effect occurred between 30–15_IFT_ testing sessions. Standardized differences in mean were calculated to determine the magnitude of the change across and between tests. According to Hopkins et al. (2001) effect size (ES) magnitudes of change were classified as trivial (>0.2), small (0.2–0.5), moderate (0.5–0.8); large (0.8–1.60), and very large (>1.60). Reliability of the change in the mean between trials was determined using intraclass correlation coefficient (ICC), typical error (TE) expressed as coefficient of variation (CV%) and smallest worthwhile change (SWC); an Excel spread sheet supplied by Hopkins (2007) was used for the calculations. ICC values of 0.1, 0.3, 0.5, 0.7, 0.9, and 1.0 were classified as low, moderate, high, very high, nearly perfect, and perfect, respectively. The following criteria was used to declare good reliability: CV < 5% and ICC > 0.69 (Buchheit et al., 2011). Appropriate performance usefulness indicators in accordance to the noise of the test result and measurement uncertainty (Hopkins, 2004) was assessed via the magnitude of the SWC. A comparison of SWC (0.2 multiplied by the between-subject SD, based on Cohen's effect size) to TE was used to establish the usefulness of a given dependent variable as follows: “Marginal” (TE > SWC), “OK” (TE = SWC) and “Good” (TE < SWC). SWC was calculated for V_IFT_, and HRpeak. Degree of coherence between VO_2max_, HRpeak and end speed of 30–15_IFT_ and CT was assessed using Pearson's product–moment correlation (*r*). Additionally, the relationship between VO_2max_ obtained from CT and V_IFT_ from 30–15_IFT_ was also investigated. Correlation values denoted association between variables and tests as small (*r* = 0.1–0.3), moderate (*r* = 0.3–0.5), large (*r* = 0.5–0.7), very large (*r* = 0.7–0.9), and almost perfect (*r* = 0.9–1.0). In a cases where small positive and negative values of confident intervals (90% CI) overlapped magnitude, the value was declared as unclear, otherwise the magnitude was deemed as observed (Hopkins, 2004). In addition, analysis of variance (2 × 2 ANOVA) was performed to determine 30–15_IFT_ performance differences between national squad (NS) and national club (NC) level players. Partial eta squared (η^2^) values of 0.02, 0.13, and 0.33 rated difference as small, moderate and high (Pierce et al., 2004). Statistical significance was indicated in cases where *p*-value was less than 0.05.

## Results

### Reliability

Similar V_IFT_ (test = 17.1 ± 1.0 km/h; retest = 17.4 ± 0.9 km/h), HRpeak (test = 196 ± 7 b.p.m; retest = 197 ± 5 b.p.m.) and VO_2max_ (test = 45.8 ± 2.8 ml/kg/min; retest = 46.5 ± 2.7 ml/kg/min) values were observed between 30–15_IFT_ testing sessions. Non-significant differences (*p* > 0.05) were observed between testing sessions for HRpeak (ES = trivial; CI 90% (−1.95; 0.82), *p* = 0.48), V_IFT_ (ES = small; CI 90% (−0.48; − 0.09), *p* = 0.23) and VO_2max_ [ES = small; CI 90% (−1.31; − 0.47), *p* = 0.20] as observed in Table 1. High test-retest reliability (ICC > 0.90; TE < 1.9%) was observed for all measures.

**Table 1:** **Reliability measure values for maximal reached speed (V_**IFT**_), peak heart rate (HRpeak) and maximal oxygen consumption (VO_**2max**_) in 30–15 intermittent fitness test**.

	**V_IFT_(km/h)**	**HRpeak (b.p.m.)**	**VO_2max_ 30–15_IFT_ (ml/kg/min)**
ES	−0.29 (Small)	−0.14 (Trivial)	−0.26 (Small)
Diff (90%CI)	0.28 (−0.48;−0.09)	<1 (−1.95; 0.82)	0.89 (−1.31; −0.47)
ICC (90%CI)	0.91 (0.80; 0.96)	0.94 (0.85; 0.97)	0.94 (0.87; 0.98)
TE (90%CI)	0.31 (0.24; 0.45)	2.0 (1.73; 3.21)	0.71 (0.55; 1.02)
CV% (90%CI)	1.8 (1.4; 2.7)	1.2 (0.9; 1.7)	1.6 (1.2; 2.3)
SWC%	0.20 (1.2%)	2.0 (0.7%)	0.55 (1.2%)
Rating	Marginal	OK	Marginal

*ES, effect size; ICC, intraclass correlation coefficient; TE, typical error of measurement; CV, Coefficient of variation; SWC, smallest worthwhile change; CI, confidence intervals*.

### Test usefulness

The TE for V_IFT_ (TE = 0.31 km/h) and VO_2max_ (TE = 0.71 ml/kg/min) was greater than the presumed SWC (SWC = 0.20 km/h and SWC = 0.55 ml/kg/min), consequently these measure were rated as “marginal.” In contrast, TE for HRpeak (~2 b.p.m) was similar to SWC and was rated as “OK.”

### Validity of the test

**Figure 1:**
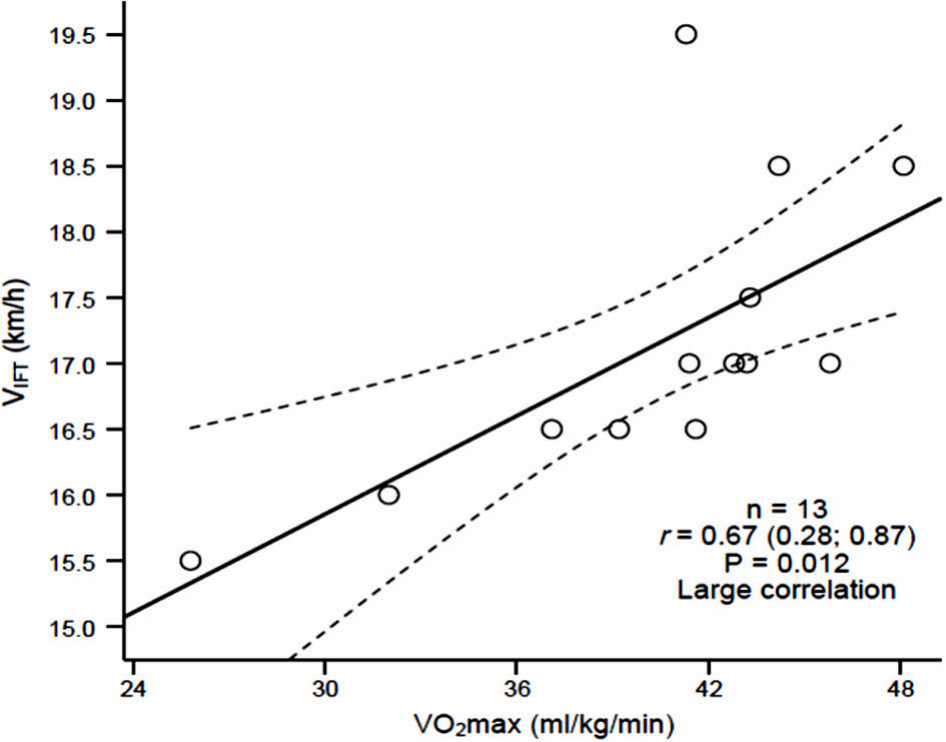
**Relationship between 30–15_**IFT**_ end speed (V_**IFT**_) and measured maximal oxygen consumption (VO_**2max**_) obtained from the incremental continuous running treadmill test**.

**Figure 2:**
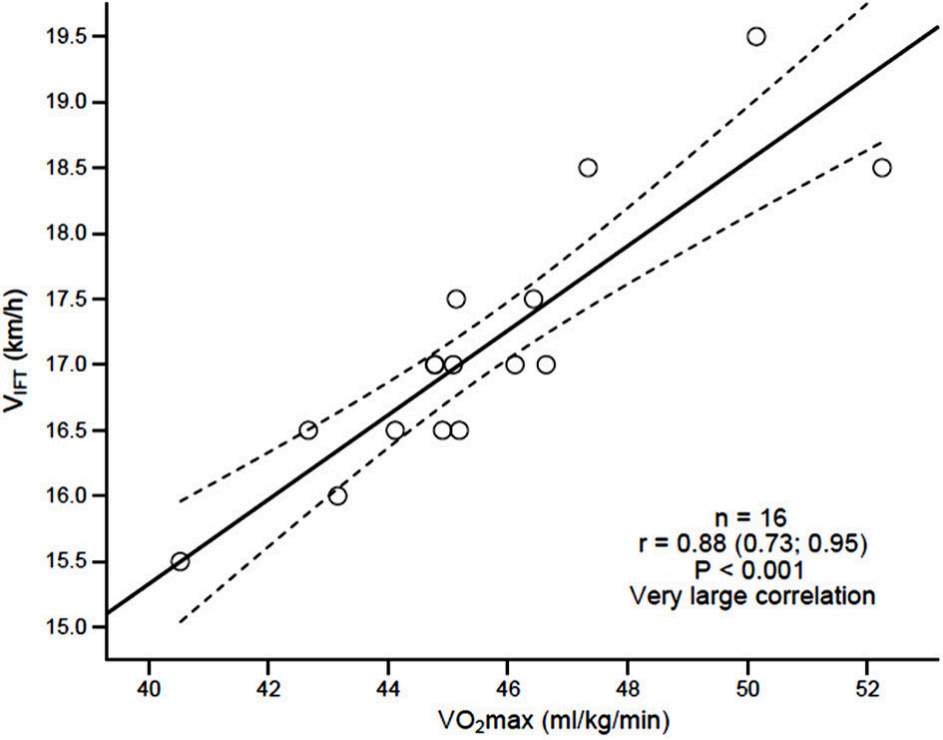
**Linear dependence of estimated maximal oxygen consumption (VO_**2**_max) based on 30–15_**IFT**_ end speed (V_**IFT**_)**.

Large to very large significant differences (*p* < 0.05) were observed between the CT and 30–15_IFT_ for VO_2max_ [ES = −1.10; *p* = 0.001; CI 90% (−4.5;−3.5)], V_CT/IFT_[ES = −0.98; *p* < 0.001; CI 90% (−7;−3)], and HRpeak (ES = −1.60; *p* < 0.001; CI 90% (−12;−7); Table 2). Large to very large correlations were observed between the CT and 30–15_IFT_ for VO_2max_(*r* = 0.67, *p* = 0.013_)_and HRpeak (*r* = 0.77, *p* = 0.02). Large to very large correlations were also observed between V_IFT_ and the following variables: V_CT_(*r* = 0.57, *p* = 0.042), CT-VO_2max_ (*r* = 0.67, *p* = 0.027; Figure 1) and 30–15_IFT_-VO_2max_ (*r* = 0.88, *p* < 0.001; Figure 2). Figure 1 explains linear relationship between maximal oxygen consumption measured directly using CT and 30–15_IFT_ end speed in 13 players and suggesting that significantly high relationship. In Figure 2, a consistent linear dependence for the maximal oxygen consumption measured indirectly from 30–15_IFT_ end speed using mathematical formula and V_IFT_ for sample of 16 players was highlighted.

**Table 2:** **Observed output for maximal oxygen consumption (VO_**2max**_) and peak heart rate (HRpeak) during 30–15 Intermittent Fitness Test (30–15_**IFT**_) and Continuous running test (CT)**.

	**CT**	**30–15_IFT_**	**Diff. (90% CI)**	**ES**	***r* (90% CI)**	**Rating**
VO_2max_	40.5±5.9	45.8±2.9^**^	5.3 (−7; −3)	−1.10	0.67^*^ (0.28; 0.87)	Large
HRpeak	185.7±5.2	195.8±7.2^**^	10.1 (−12; −7)	−1.60	0.77^**^ (0.46; 0.91)	Very large
ERV	13.2±1.2	17.1±1.0^**^	4.0 (−4.5; −3.5)	−0.98	0.57^*^ (0.13; 0.82)	Large

Data are presented as mean ± SD; CI, confidence intervals; ERV, end running velocity in km/h; ES, effect size; HRpeak, peak heart rate achieved in b.p.m.; VO_2max_, maximal oxygen uptake in ml/kg/min;

** p < 0.01,

* *p < 0.05*.

### Comparison between performance groups for 30–15_IFT_ test–retest

**Figure 3:**
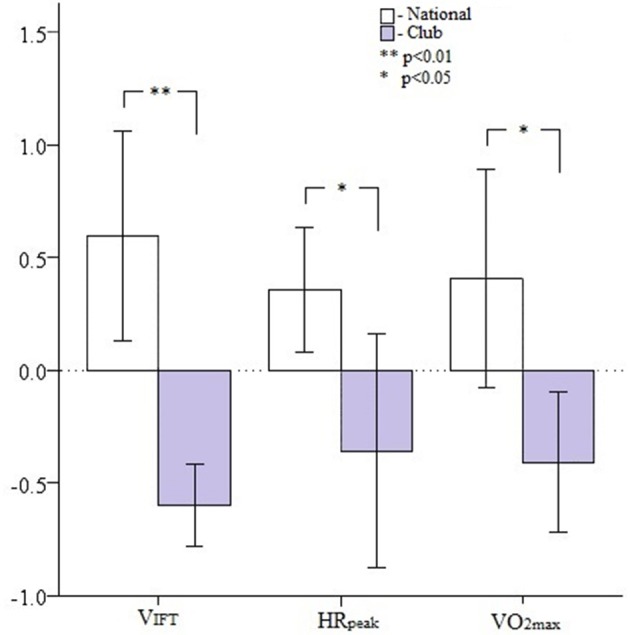
**Differences between national selection and national league level players for the 30–15_**IFT**_ test–retest end speed (V_**IFT**_), maximal heart rate (HRpeak) and maximal oxygen consumption (VO_**2max**_) expressed as standardized units (***Z***-values)**.

National squad players V_IFT_ (mean difference: 1.15 km/h; CI 90% (0.58; 1.73); *F* = 16.96, *p* < 0.001; η^2^ = 0.37), HRpeak (mean difference: 4 b.p.m; CI 90% (0.5; 8.8); *F* = 4.29, *p* = 0.048; η^2^ = 0.13) and predicted VO_2max_ (mean difference: 2.2 ml/kg/min; CI 90% (0.36; 4.0), *F* = 6.0, *p* = 0.021; η^2^ = 0.17) were significantly greater in comparison to national club level players (Table 3). Figure 3 presents a graphical interpretation of the differences between in V_IFT_, HRpeak and VO_2max_ expressed in standardized units (Z-scores) for the NS and NC level players.

**Table 3:** **Rated differences of the 30–15_**IFT**_ test–retest performance for test end speed (V_**IFT**_), heart rate peak (HRpeak) and indirect maximal oxygen consumption (VO_**2max**_) between national selection level (***n*** = 8) and national league level (***n*** = 8) players**.

	**NS**		**NC**			
	**1st trial**	**2nd trial**	**1st trial**	**2nd trial**	***F* (*p*)-value**	**Rating**
VO_2max_ (ml/kg/min)	46.7±3.0	47.5±3.0	44.4±1.9	45.4±1.8	6.0 (0.021)	High
HRpeak (b.p.m.)	199±4.0	199±4.0	194±8.0	195±6.0	4.29 (0.048)	High
V_IFT_(km/h)	17.68±1.0	18.00±1.0	16.56±0.49	16.81±0.26	16.96 (<0.001)	High

*Data are presented as mean ± SD; NS, national squad players; NC, national club league players; V_IFT_, end running velocity; HRpeak, peak heart rate; VO_2max_, maximal oxygen uptake*.

## Discussion

The aim of this study was to assess the reliability, validity and usefulness of the 30–15_IFT_ for assessing intermittent aerobic fitness in elite female soccer players. The V_IFT_ and HRpeak obtained from the 30–15_IFT_ were deemed reliable for estimating intermittent fitness capacity and HRpeak of elite female soccer players. The 30–15_IFT_ also provided a quality estimate of aerobic fitness (VO_2max_); which is in agreement with previous studies (Buchheit, 2008, 2010; Thomas et al., 2015).

The V_IFT_ reliability findings herein were (TE = 0.31 km/h, CV = 1.8%; ICC = 0.91) were similar to previous investigations; that observed low typical error (CV = 0.8 to 1.9%) in male and female team sport athletes (e.g., soccer, ice hockey, rugby and handball) (Buchheit, 2005; Buchheit et al., 2011; Scott et al., 2015; Thomas et al., 2015). A small learning effect for the 30–15_IFT_ was observed, as a “small” non-significant increase in V_IFT_ was observed from the first to the second testing session; this most likely occurred to the group's lack of experience in performing the test. Current reliability findings were also similar to other intermittent field tests, such as Yo-Yo IR1 (CV < 2.0%) (Krustrup and Bangsbo, 2001; Thomas et al., 2006) and Yo-Yo IR2 conducted on male and female team sport athletes (Thomas et al., 2006; Bangsbo et al., 2008). Based on previous research, Yo-Yo IR tests can also be used as an indicator of the intermittent aerobic fitness in elite female soccer players (Krustrup et al., 2005). In light of the fact that the Yo-Yo IR1 and 30–15_IFT_ assess different physical capacities, a large correlation (*r* = 0.75) was observed between the two intermittent fitness tests with similar levels of sensitivity following an 8 week training intervention in male soccer players (Buchheit and Rabbani, 2014). The high reliability of HRpeak (TE = 2 b.p.m; CV = 1.2%; ICC = 0.94) during the 30–15_IFT_ in elite female soccer players further supports the outcomes of previous research (Buchheit et al., 2011; Scott et al., 2015). The sample size used herein for 30–15_IFT_ test–retest reliability (*n* = 16) and validation (*n* = 13) were characterized as small; however the high reliability outcomes annulled the small sample size (Hopkins et al., 2001).

The criterion validity of the 30–15_IFT_ was assessed by comparing outcome measures to the CT (laboratory test), which is considered the “gold standard” for estimating VO_2max_. Due to relationship between HRpeak and VO_2max_ in field based tests (Scott et al., 2015) validation of 30–15_IFT_ in comparison to a CT is justified for cardiorespiratory and cardiovascular performance. Large and very large linear relationships were observed between the 30–15_IFT_ and CT for VO_2max_ (*r* = 0.67) and HRpeak (*r* = 0.77), which supports the validity of the 30–15_IFT_ for assessing maximal aerobic fitness in female soccer players. In addition, V_IFT_ was highly correlated with CT VO_2max_ (*r* = 0.67). Similar relationships between VO_2max_ and Yo-Yo IR1 performance (*r* = 0.70) (Bangsbo et al., 2008) in 141 athletes and Yo-Yo IR2 performance (*r* = 0.68) in elite female soccer players were observed (Bradley et al., 2014). Krustrup et al. (2005), observed a slightly weaker relationships (*r* = 0.55) between VO_2max_ and Yo-Yo IR1 in elite female soccer players. VO_2max_ estimated from V_*IFT*_had a very large correlation (*r* = 0.88) to CT-VO_2max_; therefore is deemed a valid and reliable alternative of predicting maximal aerobic fitness. As expected, the VO_2max_ and HRpeak values from the 30–15_IFT_ were significantly (*p* < 0.01) larger (ES > 0.8) than those values obtained from the CT. V_IFT_ obtained from the 30–15_IFT_ was 4 km/h higher than V_CT_ obtained during the CT, which is in agreement to previously predictive differences (2 to 5 km/h) (Buchheit, 2010). Current findings also support those of Buchheit (2010), implying that V_IFT_ is a valid measure of an athlete's physical fitness, and is more closely related to VO_2*max*_ and repeated intense running ability than it is to local muscular fatigue (Buchheit et al., 2011).

An intermittent fitness tests sensitivity to detect meaningful changes is vital to performance monitoring. The ability of the 30–15_IFT_ to detect meaningful changes in performance, which was assessed by comparing the TE to the SWC. Outcomes revealed that the V_IFT_ was deemed “marginally” useful, as the TE (0.31 km/h) was slightly larger than SWC (0.20 km/h); however, both the TE and SWC were lower than 0.5 km/h (one running stage), suggesting that an individual performance change as low as one stage (±0.5 km/h) to be “real and meaningful.” This is an agreement to previous findings, whom found that a 30–15_IFT_ performance change of one stage (0.5 km/h) is “meaningful” (Buchheit, 2010; Scott et al., 2015). Recommended V_IFT_ threshold values of 6–8% have been established previously as the minimal difference needed to be considered a “real” performance change for a group of athletes (Buchheit et al., 2009b,c; Buchheit et al., 2011). Furthermore, HRpeak was also deemed useful for detecting “meaningful” individual changes as small as 2 b.p.m; which is in agreement to previous findings in male rugby league players (Scott et al., 2015).

A comparison of NS and NC level players revealed significant differences in 30–15_IFT_ test–retest performance. NS players reached significantly greater V_IFT_ and HRpeak in comparison to NC players (Table 3). A mean V_IFT_ difference of 1.15 km/h was observed between groups, suggesting that there was a meaningful difference (V_IFT_ > 0.5 km/k) in 30–15_IFT_ performance between NS and NC level players. Other studies have also observed meaningful difference in 30–15_IFT_ performance (Buchheit, 2010; Scott et al., 2015). Mohr et al. (2008) and Andersson et al. (2010) between international world-class athletes and sub-elite national level athletes. These studies concluded that world-class international players performed a greater number of high-intensity running intervals during matches in comparison to their sub-elite counterparts. Since, the stage (speed) at which exhaustion occurs during incremental aerobic tests and the number of high intensity running intervals performed are highly related (*r* = 0.82) (Rampinini et al., 2007); it can be argued that 30–15_IFT_ performance may be used to differentiate between elite and sub-elite intermittent sport athletes. Future research assessing the relationships between 30–15_IFT_ performance and match kinematics (e.g., running intensity, distance covered, HR variation) in elite female intermittent team sport athletes may provide coaches with individual and positional specific diagnostics to better inform training and possibly match strategy.

In summary, the 30–15_IFT_ is reliable, valid and practically useful to assess and monitor maximal aerobic fitness (HRpeak and V_IFT_) changes in female soccer players. The current findings have provided evidence and guidelines for the meaningful detection of the intermittent fitness test performance changes. The authors suggest that further research in female soccer players focus on examining (i) the differences in 30–15_IFT_ performance based on playing position, (ii) individual differences as they relate to anthropometric and morphological characteristics, especially body mass index (Nikolaidis, 2014a) and (iii) individual and group 30–15_IFT_ performance adaptations to acute and chronic anaerobic and aerobic training.

## Conclusion

As previously iterated, the 30–15_IFT_ is a practical, valid, useful, inexpensive, and efficient aerobic intermittent field test. The test can be administered to large groups (20–30 athletes) outdoors or indoors in a relatively short amount of time (~20 min per test). Furthermore, the exhaustive sensation is lower compared to similar field tests making it useful during the preparatory (off-season and pre-season) and competitive training phases. Scientists and coaches should monitor changes in V_IFT_ to determine “meaningful” intermittent aerobic fitness changes in response to training and/or detraining. The following “meaningful” individual changes in 30–15_IFT_ performance have been proposed: 0.5 km/h (V_IFT_) and 2 b.p.m (HRpeak). A group performance change of 6–8% in V_IFT_ is required to be deemed as “real.” The 30–15_IFT_, may be more advantageous than other intermittent aerobic fitness tests in monitoring physical performance changes for intermittent sports, as is the test provides V_IFT_, HRpeak, an indirect estimate of VO_2max_ during high intensity running efforts (Thomas et al., 2015). It must be emphasized that the test is designed to accurately assess small intermittent running intensity differences (V_IFT_ changes of 0.5 km/h) and provide individualized aerobic training velocities and distances (Buchheit, 2008). It must be noted that HRpeak herein, as determined via direct and indirect aerobic fitness tests is a stable measure, and should not be confused with resting and/or submaximal heart rate variability, where day-to-day fluctuations of 10 SD units are commonly observed (Umetani et al., 1998). Due to the nature of 30–15_IFT_, the prescribed training loads (distance covered) and cardiorespiratory demands experienced by each athlete will be similar across a squad regardless of individual V_IFT_. It is also suggested that testing conditions (e.g., temperature, humidity, altitude, surface, footwear, and testing time) be controlled and standardized across testing sessions to allow for accurate performance monitoring.

## Author contributions

NČ, EJ: Substantial contributions to the conception or design of the work; HA, EK: The acquisition, analysis, or interpretation of data for the work; ZM, GS, DM: Drafting the work or revising it critically for important intellectual content; IR, DM: Final approval of the version to be published.

### Conflict of interest statement

The authors declare that the research was conducted in the absence of any commercial or financial relationships that could be construed as a potential conflict of interest.
